# Durability of Physiological Determinants of Distance‐Running Performance in Warm and Temperate Environments

**DOI:** 10.1111/sms.70394

**Published:** 2026-11-06

**Authors:** Ben Hunter, Naoto Fujii, Ed Maunder, Daniel Muniz‐Pumares

**Affiliations:** ^1^ School of Human Sciences London Metropolitan University London UK; ^2^ School of Health, Medicine, and Life Sciences University of Hertfordshire Hatfield UK; ^3^ Faculty of Health and Sport Sciences University of Tsukuba Tsukuba Japan; ^4^ School of Sport, Exercise, and Health, Faculty of Health and Environmental Sciences Auckland University of Technology Auckland New Zealand

**Keywords:** fatigue resistance, heat stress, resilience, running economy, thermoregulation, ventilatory threshold

## Abstract

Runners increasingly train and compete in warm conditions, yet it is unclear whether heat stress accelerates the deterioration of the physiological determinants of distance‐running performance during prolonged running. Ten trained runners (7 M; 3 F; 38 ± 11 years; peak oxygen uptake (V̇O_2_peak): 56.0 ± 5.7 mL·kg^−1^·min^−1^) visited the laboratory on four occasions. In randomized order, participants completed incremental tests to determine the first ventilatory threshold (VT_1_), V̇O_2_peak, and end‐test speed in temperate (TEMP; 19°C) and warm (WARM; 30°C; both 40% relative humidity) conditions in a “fresh” state (PRE). Tests were repeated (POST) after 90 min of running in the same condition at 5% Δ between the speed at VT_1_ (sVT_1_) and end‐test speed. Running economy was measured at 15‐min intervals throughout. In the rested state, sVT_1_ was ~7% (~0.8 km·h^−1^) lower in WARM than TEMP, and declined after prolonged running in both conditions (TEMP: −8.6%; WARM: −11.1%), with no condition × time interaction. V̇O_2_peak was reduced after prolonged running (TEMP: −4.8%; WARM: −4.0%), with no condition × time interaction. In contrast, running economy deteriorated to a greater extent in WARM (+5.4% vs. +1.7% from 15 to 90 min), and end‐test speed declined more in WARM than TEMP (−12.1% vs. −6.8%). We found no evidence that heat altered the durability of sVT_1_ and V̇O_2_peak when intensity was prescribed relative to condition‐specific capacity. However, sVT_1_ was already lower in WARM before prolonged running, so that after prolonged running it was, on average, 17.1% below the rested value in TEMP.

## 1 Introduction

Heat strain can impair key determinants of distance‐running performance [1]. These are typically given as maximum oxygen uptake (V̇O_2_max), running economy (RE), and the sustainable fractional utilization of V̇O_2_max [2]. The latter is typically reported as physiological transitions such as the first ventilatory threshold (VT_1_) and lactate threshold (LT). A decrease in V̇O_2_max is widely reported under heat stress, with the mechanisms well described (for a review, see Ref. [3]). Skin vasodilation and the rise in skin blood flow required for heat loss reduce central blood volume, so stroke volume falls and heart rate rises to maintain cardiac output. Maximal cardiac output, and therefore oxygen delivery to the working muscle, is consequently lower, reducing V̇O_2_max in the heat [3, 4, 5]. Importantly, sufficient initial thermal strain and increase in core temperature may be necessary before testing to observe a heat‐related reduction in V̇O_2_max, as studies using shorter incremental tests or insufficient pre‐warming have reported no change [3]. Task failure during prolonged exercise in the heat has been shown to coincide with core temperatures of ~40°C [6]. However, Ely et al. [7] demonstrate that aerobic performance can be impaired at more modest core temperatures (< 39°C) when skin temperature is high. This is because a narrower core‐to‐skin temperature gradient requires greater skin blood flow to maintain heat loss [8]. Therefore, the cardiovascular changes described above would be expected to occur at temperatures well below those associated with task failure.

In running, both the LT and V̇O_2_max have been shown to decrease by ~5% and ~8%, respectively, in 32°C and 60% relative humidity relative to cool conditions [1]. Findings at thresholds are otherwise equivocal, with unchanged LT despite greater blood lactate concentrations [9], and others demonstrating reduced cycling power output at VT_1_ in the heat [10, 11]. Downward shifts to physiological thresholds as a result of greater core temperature may be due to greater rates of carbohydrate oxidation, glycogenolysis, and lactate accumulation in hot relative to temperate or cool conditions [12, 13]. Furthermore, the accompanying rise in perceived effort and thermal strain may contribute to diminished neuromuscular function.

When considering changes to RE, James et al. [1] demonstrated improved RE during a graded exercise test in hot when compared to cool conditions. During prolonged cycling, however, a greater increase in the oxygen cost has been shown over time in hot when compared to cool conditions [14]. The differences in findings may be due to the disparate methodologies employed, with measures taken at different points of the progressive onset of heat strain and associated thermoregulatory responses. For example, when RE is measured over shorter durations in the heat, apparent improvements may reflect a greater reliance on carbohydrate oxidation, raising the ATP yield per liter of oxygen consumed [15] or improved contractile function due to increased muscle temperature [16]. Once a greater thermoregulatory demand is sustained, and core temperature exceeds ~38°C, the additional oxygen cost of hyperthermic hyperventilation [17] and of sustaining a high skin blood flow would act in the opposite direction. However, changes to RE in the heat have not been examined over longer durations. It has been posited that RE may improve because of hypohydration through sweating, and concomitant body mass loss, exacerbated through heat stress [18]. However, this has not been tested empirically.

The magnitude of the responses outlined above is dependent on how exercise intensity is prescribed across conditions. For example, Charoensap et al. [19] matched exercise intensity to 95% of the heart rate associated with VT_1_ across 18°C and 33°C, observing lower carbohydrate oxidation in the heat alongside reduced power output and heat production. Anchoring intensity to condition‐specific capacity, therefore, lowers the absolute workload, affecting the strain imposed. Studies that match absolute speed or power impose a higher relative intensity in the heat and are likely to magnify the apparent deterioration in the determinants of performance. Studies anchored to condition‐specific capacity hold relative intensity broadly constant but reduce the external demand and the associated thermal load. The two approaches address different questions, which is an important consideration when comparing findings across studies.

Irrespective of environmental conditions, physiological factors associated with distance running deteriorate during exercise [20, 21, 22, 23]. The ability to resist deterioration to these factors following prolonged exercise, defined herein as durability [24], has been recently proposed [25] and subsequently evidenced [21], as an independent determinant of distance‐running performance. Prolonged exercise elevates core temperature irrespective of ambient conditions, so if the deterioration in physiological function after prolonged exercise is partly thermally mediated, the additional thermal load of a warm environment would be expected to exacerbate it, even when relative intensity is matched. This possibility has not been examined directly, as no study has quantified the determinants of distance‐running performance following prolonged exercise in the heat. The purpose of this study was to examine whether heat stress negatively impacts the durability of the primary determinants of long‐distance running performance during relative‐intensity‐matched running bouts. It was hypothesized that the durability of V̇O_2_peak, RE, and VT_1_ would be impaired when exercise was performed in warm conditions relative to temperate conditions.

## 2 Materials and Methods

### 2.1 Participants

Fifteen non‐heat‐acclimated participants gave voluntary written informed consent to take part in the study after the experimental procedures, risks, and potential benefits had been explained. Participants reported no travel to or residence in a hot climate, and no heat acclimation or sauna protocol in the preceding 4 weeks. Testing took place during the UK summer, and we cannot exclude some degree of seasonal heat acclimatization, which has been reported in temperate summers of comparable severity [26]. As all trials were counterbalanced and each participant completed all conditions within 4 weeks, any adaptation seems an unlikely explanation for the differences observed between environments. All participants were in good health, had no recent history of lower limb injuries (< 3 months), and were habituated to running (currently running at least 3 h·week^−1^). Participants were also required to demonstrate a recent running performance of 10 km in < 45 min or 5 km in < 22 min (10 km in < 52:30 min or 5 km in < 25 min for female participants) and have run for at least 90 min during at least one session in the past month. The performance inclusion criteria for female participants were originally set at a 10‐km time of < 50 min or a 5‐km time of < 24 min to provide comparable age‐graded standards between sexes. Following limited recruitment of female participants, these criteria were subsequently relaxed to < 52:30 min and < 25 min, respectively. Ethical approval was granted by the University of Hertfordshire Health, Science, Engineering & Technology Ethics Committee with Delegated Authority (Protocol: 0922 2025 Jun HSET). All methods were conducted in line with the Code of Ethics of the World Medical Association and Declaration of Helsinki, except for pre‐registration.

Participants were required to complete food diaries in the 24 h prior to each visit and were instructed to repeat the reported food and fluid intake before each visit. Participants were asked to abstain from caffeine for a minimum of 6 h prior to each visit, to avoid alcohol for 24 h prior to each visit, and to avoid strenuous exercise in the 24 h prior to each visit. For female participants, menstrual cycle phase, hormonal contraceptive use, or timing of testing were not controlled. Participants were asked to use the same footwear for each visit and were not permitted to use carbon fiber plate shoes. In addition, participants were asked to wear similar clothing for each visit. A priori sample size estimation suggested that a sample of 7 participants would be sufficient to detect a medium effect of time (*f* = 0.66) [22] and heat (*f* = 0.33) [1] on the moderate‐to‐heavy intensity transition with statistical power of 0.8 and alpha of 0.05, with a high test–retest reliability (ICC > 0.91). This calculation was used to support study feasibility, but it was not an interaction‐specific power calculation for the condition × time effect used to evaluate durability, as this has not been reported previously. Consequently, non‐significant interaction effects should be interpreted cautiously, particularly for modest between‐condition differences.

### 2.2 Experimental Design

All trials were conducted on the same treadmill (Pulsar, h/p/cosmos Sport and Medical, Traunstein, Germany) set to a gradient of 1% [27]. The study consisted of two testing blocks totalling four visits (Figure 1), with characterization trials in Block 1 separated by a minimum of 24 h, and a minimum of 72 h between the prolonged trials in Block 2. The first block consisted of two characterization trials in either temperate (TEMP; 19°C and 40% relative humidity) or warm (WARM; 30°C and 40% relative humidity) conditions on separate visits. The second block consisted of two characterization tests following a prolonged running trial performed in either TEMP or WARM. Testing sessions occurred over a maximum of 4 weeks and took place at approximately (±2 h) the same time of day. The conditions used during the visits in each block were completed in a randomized and counterbalanced order, and controlled using an environmental chamber (Sporting Edge, Basingstoke, UK). Trials within Block 1 preceded trials within Block 2, but visits within each block were randomized. Although previous studies have employed more severe heat stress (e.g., Refs [1, 19]), a moderate heat stress was chosen as it: (i) aligned better with WBGT values that could be anticipated during spring or autumn marathons, and (ii) allowed for a majority of participants to complete the prolonged run in WARM whilst limiting the risk of exceeding the temperature cut‐off of 39.5°C.

**FIGURE 1:**
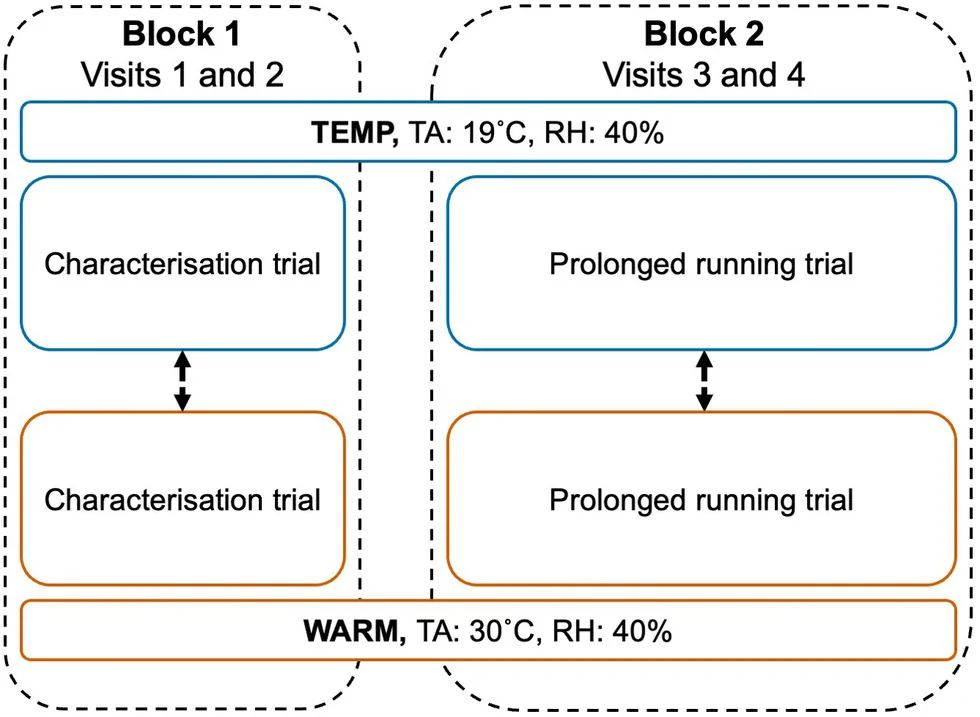
Experimental design. Block 1 consisted of two characterization trials, and Block 2 comprised two prolonged running trials. The condition order was randomized (vertical arrows) within each block. RH, relative humidity; TA, ambient temperature; TEMP, temperate condition; WARM, warm condition.

### 2.3 Characterization Trials

The characterization trials consisted of an incremental exercise test on a treadmill to task failure to determine V̇O_2_peak and VT_1_, and the speed associated with each. Participants entered the chamber and immediately (within a maximum of 2 min) began the incremental exercise test which commenced at a speed commensurate with fitness levels (TEMP PRE: 9.1 ± 0.7 km·h^−1^; TEMP POST: 8.7 ± 1.0 km·h^−1^; WARM PRE: 9.0 ± 0.7 km·h^−1^; WARM POST: 8.5 ± 1.0 km·h^−1^) and increased by 1 km·h^−1^ every 3 min. Following the consistent attainment of a respiratory exchange ratio of 1.0 and a clear rise in V̇_E_/V̇O_2_, the treadmill speed increased by 1 km·h^−1^ every minute until task failure. Pulmonary gas exchange data and heart rate data were collected throughout the incremental exercise test. V̇O_2_peak was determined as the highest 30‐s rolling mean. VT_1_ was given as the V̇O_2_ at which a rise in V̇_E_/V̇O_2_ and end‐tidal PO_2_ without a concomitant increase in V̇_E_/V̇CO_2_ occurred. This was agreed independently by two researchers. The speed at which VT_1_ (sVT_1_) occurred was calculated by plotting V̇O_2_ from the final 30 s of each 3‐min step against speed. The end‐test speed was given as the speed of the last stage of the incremental exercise test fully completed. If the final stage was not completed in full, end‐test speed was calculated as:

$$\text{End‐test speed}=\mathrm{LCS}+\left(\frac{t}{60\;s}\right)\times 1\;\mathrm{km}\cdot h^{-1}$$

where LCS represents the last completed stage (km·h^−1^), *t* is the time of the incomplete stage performed, 60 s refers to the step duration in seconds, and 1 km·h^−1^ denotes the delta speed from the previous stage.

### 2.4 Prolonged Running Trials

Prior to commencement of prolonged running trials, hydration status was assessed using a handheld digital refractometer (Pocket Osmocheck, Vitech Scientific, UK). Where urine osmolality was > 700 mOsm·kg^−1^ H_2_O, participants were required to drink 300 mL of plain water. Participants began the prolonged running trial within 2 min of entering the environmental chamber. The prolonged run was set at a condition‐specific speed corresponding to 5% Δ, where Δ represents the difference between the sVT_1_ and end‐test speed for the respective temperatures. This intensity was chosen based on the findings of Carter et al. [28], where the critical speed occurred at ~40% Δ, so that the participants were exercising towards the lower end of the heavy domain in the current study. Each prolonged run was followed by a 5‐min rest, completed in the same environmental condition, after which participants commenced an incremental exercise test which was carried out in accordance with the characterization trials (POST). Where necessary, the starting speed of the POST incremental exercise test was adjusted downwards to allow for a baseline of submaximal stages below the expected sVT_1_, so that VT_1_ and the speed at which it occurred could still be determined. Physiological measures, core and skin temperature, and perceptual measures were recorded at baseline and at 15‐min intervals thereafter corresponding to 15, 30, 45, 60, 75, and 90 min. Breath‐by‐breath gas exchange data and heart rate were collected for 5 min preceding each interval, with the final minute of each collection used for further analysis. Perceptual measures were recorded 1 min prior to each interval. Participants were allowed to consume water *ad libitum* throughout each prolonged run [29]. Nude body mass was recorded pre‐ and post‐exercise, and water intake was measured to account for sweat loss. Participants were instructed to towel off any excess sweat post‐exercise.

### 2.5 Measures

#### 2.5.1 Physiological Measures

During each visit, participants were fitted with an oronasal mask (7450 V2, Hans Rudolph, Kansas City, MO). Expired gas volume and concentration were sampled continuously (MetaLyzer 3B, Cortex Biophysik, Leipzig, Germany). The MetaLyzer 3B has previously demonstrated excellent inter‐session reliability [30], with relatively low error in warm conditions [31]. Following prolonged running, the between‐day typical error for relative V̇O_2_ at VT_1_ and sVT_1_ is ~1.9 mL·kg^−1^·min^−1^ and ~0.5 km·h^−1^, respectively. The percentage deterioration in sVT_1_, which is how durability was quantified here, has shown poor reliability (ICC ~0.07–0.25; typical error ~3.6–3.9 percentage points; [32]). Prior to each visit, the gas analyzer was calibrated with a gas of known concentration and the turbine volume transducer was calibrated using a 3‐L syringe (Hans Rudolph, Kansas City, MO). Pulmonary gas exchange was measured breath‐by‐breath. Prior to data extraction, gas exchange data were filtered using custom‐written scripts in MATLAB 2023b (Mathworks, Natick, MA). First, errant breaths, defined as those lying 2 SD away from the local mean of 5 breaths, were removed and linearly interpolated. Data were subsequently filtered with a third‐order Butterworth low‐pass filter (cut‐off 0.04 Hz) applied in zero‐phase (forward‐reverse) to avoid phase distortion [33]. Energy expenditure (EE), fat oxidation, and carbohydrate oxidation rates were calculated in line with Jeukendrup and Wallis [15] where:

$$\text{Energy expenditure}\;\left(\text{kcal}\cdot \min^{-1}\right)=\left(0.550\times \dot{V}CO_{2}\right)+\left(4.471\times \dot{V}O_{2}\right)$$

$$\begin{array}{c} \mathrm{Fat}\;\text{oxidation}\;\left(g\cdot \min^{-1}\right)=\left(1.695\times \dot{V}O_{2}\right)-\left(1.701\times \dot{V}CO_{2}\right) \end{array}$$

$$\text{Carbohydrate oxidation}\;\left(g\cdot \min^{-1}\right)=\left(4.210\times \dot{V}CO_{2}\right)-\left(2.962\times \dot{V}O_{2}\right)$$

RE was expressed as the oxygen cost of running (mL·kg^−1^·km^−1^), calculated as V̇O_2_ (mL·kg^−1^·min^−1^) divided by running speed (km·min^−1^). The energetic cost of running (EC; kcal·kg^−1^·km^−1^) was calculated in the same way from energy expenditure. Changes in body mass due to sweat were used to adjust RE and EC measured during the prolonged trials. Individual linear regressions (intercept as the pre‐body mass and the slope given as the loss of body mass over time) were calculated for each participant and each visit based on the pre‐ and post‐exercise measurements. Post‐exercise measurements were corrected for fluid ingestion by subtracting the mass of the fluid ingested throughout the visit. Body mass was then linearly interpolated for each time point throughout the prolonged trials to correct RE and EC. Since the slope and intercept differed between participants and visits, a single equation was not applied across the sample. V̇O_2_peak was given as the highest rolling 30‐s mean and expressed in absolute terms and relative to body mass. Heart rate (HR) data were recorded every 1 s during periods where pulmonary gases were collected (H10, Polar Electro Oy, Kempele, Finland).

#### 2.5.2 Core and Skin Temperature

To record gastrointestinal temperature (*T*_core_), participants ingested a radiotelemetry pill (e‐Celsius Performance, BodyCap, Hérouville Saint‐Clair, France). To avoid artifacts caused by water consumption, and to ensure passage into the gastrointestinal tract prior to testing, participants were instructed to ingest the pill at least 6 h prior to arrival [34]. The time of ingestion relative to the prolonged trial was kept consistent within participants. To mitigate against potential artifacts caused by drinking cold water, beverage temperature was maintained at ~37°C using a bottle warmer (Avent, Philips, Amsterdam, Netherlands). Wireless skin thermochrons (eTemp Performance, BodyCap, Hérouville Saint‐Clair, France) were attached to four sites (chest, lateral deltoid, quadriceps, and gastrocnemius) to measure skin temperature. Mean skin temperature (*T*_skin_) was calculated as: *T*_skin_ = 0.3 × (chest + lateral deltoid) + 0.2 × (quadriceps + gastrocnemius) [35]. *T*_core_ and *T*_skin_ data were sampled continuously throughout, with 5‐min bin averages at 15‐min intervals exported for further analysis. Mean body temperature (*T*_mean_) was calculated as: 0.8 × *T*_core_ + 0.2 × *T*_skin_, and the core‐to‐skin temperature gradient was given as the difference between *T*_core_ and *T*_skin_. To adhere to local health and safety measures, exercise was terminated if *T*_core_ reached 39.5°C. No participants exceeded this threshold.

#### 2.5.3 Perceptual Measures

Rating of perceived exertion for legs (RPE_leg__s_) and overall (RPE_O_) were used to test for local and global perceptions of exertion [36]. The modified Borg dyspnoea scale was used to assess difficulty of breathing using a scale from 0 to 10, where 0 represents no dyspnoea and 10 represents maximal dyspnoea [37]. Perceived thermal comfort on a 1‐to‐10 scale and thermal sensation on a 1‐to‐14 scale were adapted from Gagge et al. [38].

### 2.6 Statistical Analysis

Jamovi statistical software (Version 2.3.28.0) was used for all statistical analyses. All data are presented as mean ± SD. Statistical significance was set at an *α* level of 0.05. Normality of the data was confirmed using the Shapiro–Wilk test. Where the assumption of sphericity was not met, Huynh‐Feldt adjustments were made. The effects of temperature (TEMP and WARM) and time (PRE and POST) on speed, V̇O_2_, HR, and EE at VT_1_, and V̇O_2_peak, were each assessed using a two‐way repeated measures ANOVA. Durability was defined as the change in each physiological determinant from PRE to POST, except for the case of RE, where the change from the first 15‐min interval of the prolonged trial was used as a baseline for comparison at later time points. Therefore, condition × time interaction effects were used to determine whether the magnitude of deterioration differed between TEMP and WARM. An additional descriptive two‐way repeated measures ANOVA was used to test for effects of temperature (TEMP and WARM) and time for physiological variables, perceptual variables (RPE_legs_, RPE_O_, thermal comfort, thermal sensation, and dyspnoea), *T*_skin_, *T*_core_, *T*_mean_, and core‐to‐skin temperature gradient. Bonferroni post hoc analysis was used to explore main effects and interaction effects. No artificial intelligence (AI) or machine‐learning tools, including generative AI systems, were used to assist with study procedures, scripting, data cleaning, statistical analysis, literature searches, figure preparation, manuscript drafting or editing, or responses to reviewers.

## 3 Results

Of the 15 participants who started the study, 10 runners (7 M; 3 F; 38 ± 11 years; 1.74 ± 0.11 m; 68.2 ± 13.8 kg) completed all laboratory visits. Two runners could not complete the prolonged WARM trial, one runner withdrew due to injury, one runner withdrew due to illness, and the dataset from one participant was withdrawn due to a technical fault. Both participants who stopped the WARM trial early told investigators that they felt generally unwell and that their legs felt heavy, and each asked to withdraw. Neither described anything similar during TEMP. These comments were not recorded formally and are reported here for context only.

### 3.1 Prolonged Trials

The TEMP speed at VT_1_ was 11.5 ± 0.7 km·h^−1^ and the WARM speed at VT_1_ was 10.7 ± 1.1 km·h^−1^ (*p* < 0.001). End‐test speeds were 17.0 ± 1.4 and 16.7 ± 1.4 km·h^−1^ for TEMP and WARM, respectively (*p* = 0.631). Therefore, during the prolonged trials, participants ran at 11.8 ± 0.7 and 11.0 ± 1.1 km·h^−1^ in TEMP and WARM trials, covering 17.7 ± 1.1 and 16.5 ± 1.6 km, respectively (*p* = 0.002). The relative intensity at 15 min did not differ (*p* = 0.520) between TEMP (103.4% ± 4.1% of V̇O_2_ at VT_1_) and WARM (102.1% ± 3.0% of V̇O_2_ at VT_1_) conditions. Changes in body mass were greater in WARM (−3.5% ± 0.9%; −2.4 ± 0.9 kg) when compared to TEMP (−2.7% ± 0.8%; −1.8 ± 0.6 kg; *p* = 0.021). The duration of the incremental test was longer during PRE compared to POST (*p* < 0.001) in both TEMP (21.9 ± 2.9 min vs. 18.3 ± 1.4 min) and WARM (21.6 ± 3.2 min vs. 17.5 ± 1.8 min), with no differences between conditions (*p* = 0.356).

Figure 2 shows the thermoregulatory responses across conditions. During the prolonged trials, greater peak *T*_core_ (WARM: 38.9°C ± 0.5°C vs. TEMP: 38.3°C ± 0.3°C; *p* = 0.004), peak *T*_skin_ (WARM: 35.0°C ± 0.5°C vs. TEMP: 31.6°C ± 1.9°C; *p* < 0.001), and peak *T*_mean_ (WARM: 38.0°C ± 0.5°C vs. TEMP: 36.8°C ± 0.4°C; *p* < 0.001) were evident in WARM compared to TEMP. In contrast, a lower peak core‐to‐skin temperature gradient was observed in WARM compared to TEMP (WARM: 4.4°C ± 1.2°C vs. TEMP: 7.8°C ± 1.5°C; *p* < 0.001). There was a significant interaction between time and condition for *T*_core_ (*F*_1.75,15.72_ = 11.62, *p* = 0.001, $\eta_{p}^{2}$ = 0.56), *T*_skin_ (*F*_2.21,19.89_ = 6.75, *p* = 0.005, $\eta_{p}^{2}$ = 0.43), *T*_mean_ (*F*_1.94,17.47_ = 16.49, *p* < 0.001, $\eta_{p}^{2}$ = 0.70), but not core‐to‐skin temperature gradient (*F*_2.17,19.55_ = 2.92, *p* = 0.073, $\eta_{p}^{2}$ = 0.25). There were significant effects of time for all temperature parameters (all *p* ≤ 0.02) apart from *T*_skin_. In addition, there were significant effects of condition for *T*_skin_, *T*_mean_, and core‐to‐skin temperature gradient (all *p* < 0.001), but not *T*_core_ (*p* = 0.114). Pairwise comparisons are given in Figure 2.

**FIGURE 2:**
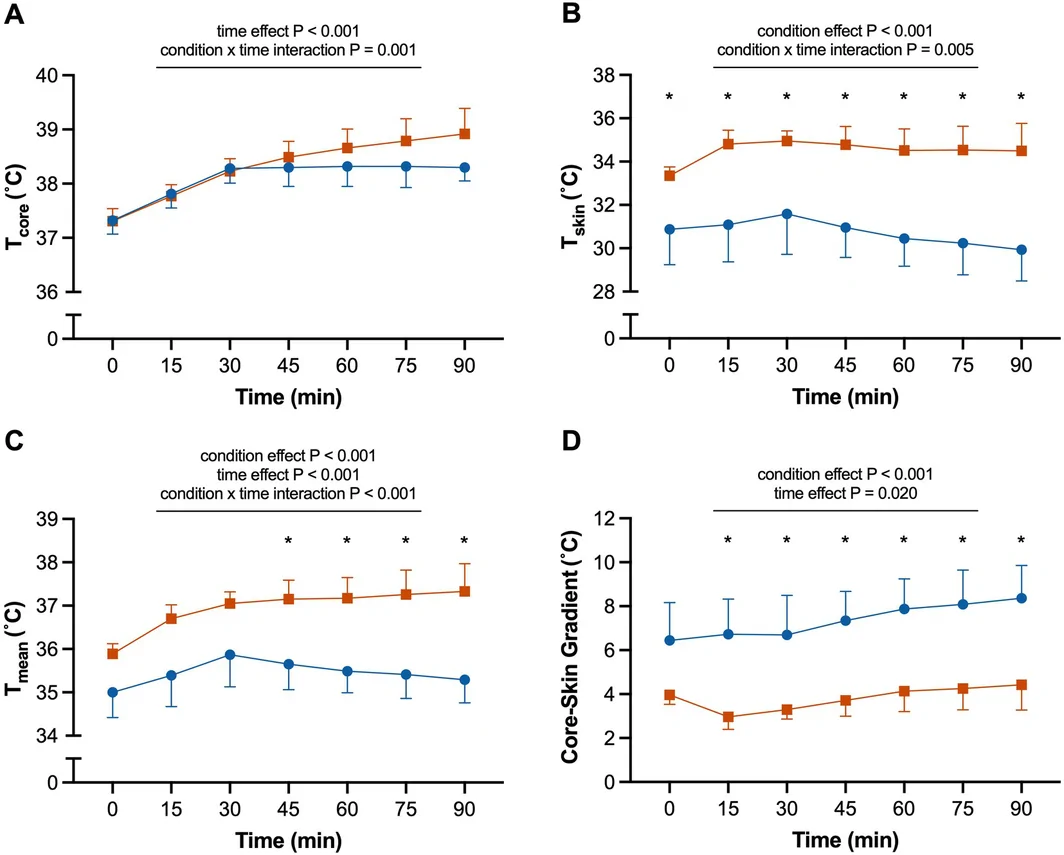
Thermoregulatory responses to the prolonged trial. (A) Gastrointestinal temperature (*T*_core_); (B) skin temperature (*T*_skin_); (C) mean body temperature (*T*_mean_); (D) core‐to‐skin temperature gradient. Orange squares indicate the WARM condition, and blue circles indicate the TEMP condition. * denotes significant difference between conditions at that time point *p* < 0.05. Data are mean ± SD. *n* = 10.

During the prolonged trials, there was a significant effect of time on RE (*F*_5,45_ = 4.71, *p* = 0.002, $\eta_{p}^{2}$ = 0.34) and EC (*F*_5,45_ = 3.51, *p* = 0.009, $\eta_{p}^{2}$ = 0.28), as well as interaction effects between time and condition on both RE (*F*_5,45_ = 2.54, *p* = 0.042, $\eta_{p}^{2}$ = 0.22) and EC (*F*_5,45_ = 2.43, *p* = 0.049, $\eta_{p}^{2}$ = 0.21). However, no significant differences from the 15‐min baseline were detected when applying post hoc corrections (Figure 3). Furthermore, no differences were evident between the conditions (both *p* ≥ 0.149). No significant differences in carbohydrate or fat oxidation rates were shown between conditions, with no significant time × condition interaction. However, there was a significant effect of time on carbohydrate oxidation rates (*F*_5,45_ = 37.57, *p* < 0.001, $\eta_{p}^{2}$ = 0.81) and fat oxidation rates (*F*_5,45_ = 30.80, *p* < 0.001, $\eta_{p}^{2}$ = 0.77), with significant differences noted from 30 min onwards relative to the 15‐min baseline (Figure 3). Total metabolic energy expenditure during the 90‐min run was significantly greater in TEMP compared to WARM (1300 ± 223 kcal vs. 1228 ± 196 kcal, *t*(9) = 3.04, *p* = 0.014, *d* = 0.96). However, no significant difference was present in the total fat oxidation (73 ± 12 g vs. 71 ± 15 g, *t*(9) = 0.35, *p* = 0.732, *d* = 0.11) or carbohydrate oxidation (146 ± 50 g vs. 131 ± 45 g, *t*(9) = 1.07, *p* = 0.311, *d* = 0.34).

**FIGURE 3:**
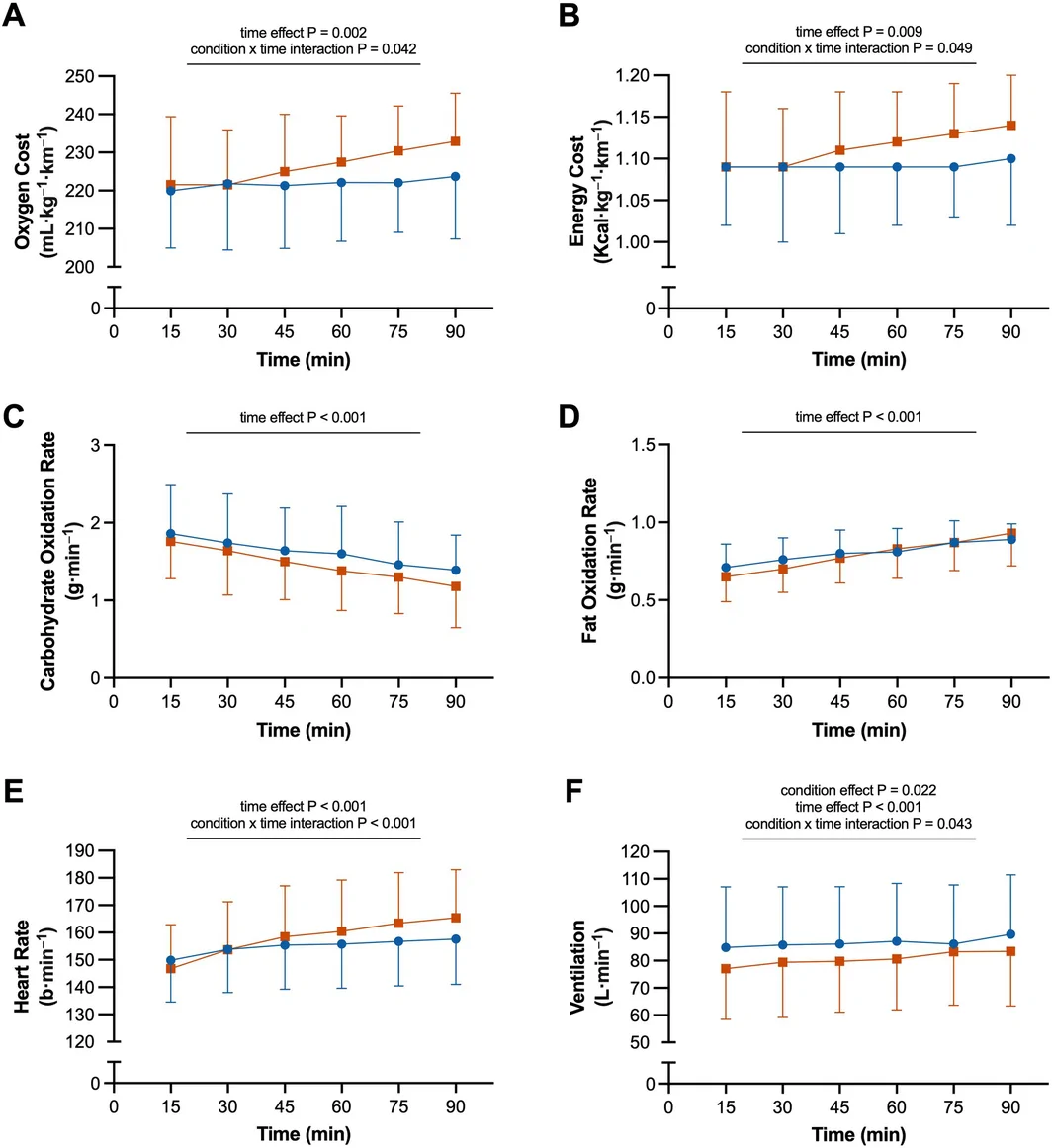
Physiological responses to prolonged trials. (A) Running economy, expressed as oxygen cost (mL·kg^−1^·km^−1^); (B) energetic cost of running (kcal·kg^−1^·km^−1^); (C) carbohydrate oxidation rate (g·min^−1^); (D) fat oxidation rate (g·min^−1^); (E) heart rate (b·min^−1^); (F) minute ventilation (L·min^−1^). Orange squares indicate the WARM condition, and blue circles indicate the TEMP condition. Data are mean ± SD. *n* = 10.

There was a significant effect of time on heart rate (*F*_1.59,14.30_ = 37.77, *p* < 0.001, $\eta_{p}^{2}$ = 0.81) and minute ventilation (*F*_4.44,39.92_ = 12.49, *p* < 0.001, $\eta_{p}^{2}$ = 0.58). Furthermore, there were significant time × condition interactions shown for both heart rate (*F*_5,45_ = 57.69, *p* < 0.001, $\eta_{p}^{2}$ = 0.87) and minute ventilation (*F*_5,45_ = 2.51, *p* = 0.043, $\eta_{p}^{2}$ = 0.22). Following post hoc analysis, compared to 15 min, heart rate exhibited changes from baseline at 30, 45, and 60 min in TEMP, whereas heart rate showed a sustained increase from 30 min onwards in WARM. Minute ventilation was greater in TEMP when compared to WARM (*F*_1,9_ = 7.64, *p* = 0.022, $\eta_{p}^{2}$ = 0.46).

Table 1 shows perceptual responses during the prolonged trials across both conditions. Across the prolonged trials, there were significant effects of time on all perceptual responses, including RPE_O_ (*F*_2.20,19.82_ = 24.65, *p* < 0.001, $\eta_{p}^{2}$ = 0.73), RPE_legs_ (*F*_2.05,18.44_ = 16.46, *p* < 0.001, $\eta_{p}^{2}$ = 0.65), dyspnoea (*F*_2.42,21.75_ = 9.57, *p* < 0.001, $\eta_{p}^{2}$ = 0.52), thermal comfort (*F*_5,45_ = 9.39, *p* < 0.001, $\eta_{p}^{2}$ = 0.51), and thermal sensation (*F*_3.06,27.53_ = 10.09, *p* < 0.001, $\eta_{p}^{2}$ = 0.53). In contrast, condition had no significant effect on RPE_O_, RPE_legs_, or dyspnoea (all *p* ≥ 0.152), but did significantly affect thermal comfort (*F*_1,9_ = 15.58, *p* = 0.003, $\eta_{p}^{2}$ = 0.63) and thermal sensation (*F*_1,9_ = 65.85, *p* < 0.001, $\eta_{p}^{2}$ = 0.88), with consistently higher ratings in the WARM condition. There were no significant time × condition interactions for any perceptual measure (all *p* ≥ 0.067).

**TABLE 1:** Ratings of whole‐body perceived exertion (RPE_O_), ratings of perceived exertion at the legs (RPE_legs_), thermal perceptions, and dyspnoea during prolonged running in temperate (TEMP) and warm (WARM) environmental conditions.

		Time point						ANOVA results		
Variable	Condition	15 min	30 min	45 min	60 min	75 min	90 min	Condition	Time	Time × condition
RPE_O_	TEMP	11.6 ± 0.7	11.9 ± 0.9	12.2 ± 0.9	12.5 ± 1.1	13.1 ± 1.6	13.5 ± 1.6	*F*_(1,9)_ = 0.97 *p* = 0.351 $\eta_{p}^{2}$ = 0.10	***F***_**(2.20,19.82)**_ **= 24.65** ***p* < 0.001** $\eta_{p}^{2}$ **= 0.73**	*F*_(3.91,35.18)_ = 1.36 *p* = 0.267 $\eta_{p}^{2}$ = 0.13
	WARM	11.1 ± 0.9	11.6 ± 1.3	11.7 ± 1.3	12.3 ± 1.7	12.5 ± 1.4	13.6 ± 1.6			
RPE_legs_	TEMP	11.7 ± 0.5	12.1 ± 0.9	12.3 ± 1.2	12.5 ± 1.2	13.2 ± 1.7	13.4 ± 1.8	*F*_(1,9)_ = 2.45 *p* = 0.152 $\eta_{p}^{2}$ = 0.21	***F***_**(2.05,18.44)**_ **= 16.46** ***p* < 0.001** $\eta_{p}^{2}$ **= 0.65**	*F*_(2.02,18.15)_ = 2.52 *p* = 0.108 $\eta_{p}^{2}$ = 0.22
	WARM	10.8 ± 1.5	11.2 ± 1.3	11.8 ± 1.4	12.3 ± 1.6	12.6 ± 1.2	13.6 ± 1.7			
Thermal comfort	TEMP	1.9 ± 1.0	2.6 ± 0.5	2.6 ± 0.5	2.7 ± 0.5	2.9 ± 0.3	2.8 ± 0.4	***F***_**(1,9)**_ **= 15.58** ***p* = 0.003** $\eta_{p}^{2}$ **= 0.63**	***F***_**(5,45)**_ **= 9.39** ***p* < 0.001** $\eta_{p}^{2}$ **= 0.51**	*F*_(5,45)_ = 1.46 *p* = 0.222 $\eta_{p}^{2}$ = 0.14
	WARM	2.7 ± 0.7	3.0 ± 0.5	3.0 ± 0.5	3.1 ± 0.6	3.2 ± 0.6	3.5 ± 0.5			
Thermal sensation	TEMP	3.2 ± 0.8	3.7 ± 0.5	4.0 ± 0.7	4.0 ± 0.7	4.1 ± 0.7	4.2 ± 0.6	***F***_**(1,9)**_ **= 65.85** ***p* < 0.001** $\eta_{p}^{2}$ **= 0.88**	***F***_**(3.06,27.53)**_ **= 10.09** ***p* < 0.001** $\eta_{p}^{2}$ **= 0.53**	*F*_(5,45)_ = 2.23 *p* = 0.067 $\eta_{p}^{2}$ = 0.20
	WARM	4.7 ± 0.5	4.8 ± 0.4	4.9 ± 0.3	5.0 ± 0.0	5.3 ± 0.5	5.6 ± 0.5			
Dyspnoea	TEMP	1.7 ± 1.1	1.7 ± 1.1	2.3 ± 1.2	2.4 ± 1.2	2.5 ± 1.1	2.6 ± 1.3	*F*_(1,9)_ = 1.97 *p* = 0.194 $\eta_{p}^{2}$ = 0.18	***F***_**(2.42,21.75)**_ **= 9.57** ***p* < 0.001** $\eta_{p}^{2}$ **= 0.52**	*F*_(5,45)_ = 1.28 *p* = 0.288 $\eta_{p}^{2}$ = 0.12
	WARM	1.4 ± 1.2	1.6 ± 1.1	1.8 ± 0.9	1.9 ± 1.1	2.1 ± 1.3	2.5 ± 1.4			

*Note:* Data are presented as mean ± SD. ANOVA results given in bold denote significant effects or interactions (*p* < 0.05). *n* = 10.

### 3.2 Moderate‐To‐Heavy Intensity Transition

There was a significant effect of condition (*F*_1,9_ = 43.80, *p* < 0.001, $\eta_{p}^{2}$ = 0.83) and time (*F*_1,9_ = 26.68, *p* < 0.001, $\eta_{p}^{2}$ = 0.75) on the speed at VT_1_, with lower speeds observed in WARM vs. TEMP, and post vs. pre (Figure 4; Table 2). There was a significant effect of condition on absolute V̇O_2_ at VT_1_ (*F*_1,9_ = 20.01, *p* = 0.002, $\eta_{p}^{2}$ = 0.69) and relative V̇O_2_ at VT_1_ (*F*_1,9_ = 17.82, *p* = 0.002, $\eta_{p}^{2}$ = 0.66), with lower V̇O_2_ values at VT_1_ in WARM vs. TEMP (Figure 5; Table 2). Absolute V̇O_2_ values at VT_1_ were significantly lower in post vs. pre (*F*_1,9_ = 23.71, *p* < 0.001, $\eta_{p}^{2}$ = 0.72); however, no differences were detected in relative V̇O_2_ at VT_1_ (*F*_1,9_ = 4.73, *p* = 0.058, $\eta_{p}^{2}$ = 0.34). No significant condition × time interaction effects were evident in speed and absolute or relative V̇O_2_ at VT_1_ (all *p* ≥ 0.312). The between‐condition difference in the magnitude of the sVT_1_ decline (WARM − TEMP) was −0.21 km·h^−1^ (95% CI −0.65 to +0.23 km·h^−1^). With the present sample, the smallest between‐condition difference detectable with 80% power was 0.61 km·h^−1^. There was a significant effect of time (*F*_1,9_ = 24.48, *p* < 0.001, $\eta_{p}^{2}$ = 0.73) and condition (*F*_1,9_ = 12.16, *p* = 0.007, $\eta_{p}^{2}$ = 0.57) on metabolic energy expenditure at VT_1_, but no condition × time interaction (*F*_1,9_ = 0.08, *p* = 0.789, $\eta_{p}^{2}$ = 0.01). Specifically, lower metabolic energy expenditure at VT_1_ was observed in WARM compared to TEMP and in post compared to pre (Table 2). Significant effects of condition were observed in minute ventilation (*F*_1,9_ = 15.78, *p* = 0.003, $\eta_{p}^{2}$ = 0.64), and RER (*F*_1,9_ = 5.88, *p* = 0.038, $\eta_{p}^{2}$ = 0.39), as well as effects of time for minute ventilation (*F*_1,9_ = 16.61, *p* = 0.003, $\eta_{p}^{2}$ = 0.65), and RER (*F*_1,9_ = 31.2, *p* < 0.001, $\eta_{p}^{2}$ = 0.78; Table 2). No significant interaction effects were shown for minute ventilation or RER. Heart rate showed no significant differences between time points or conditions, and no interaction effects were evident (all *p* ≥ 0.121).

**FIGURE 4:**
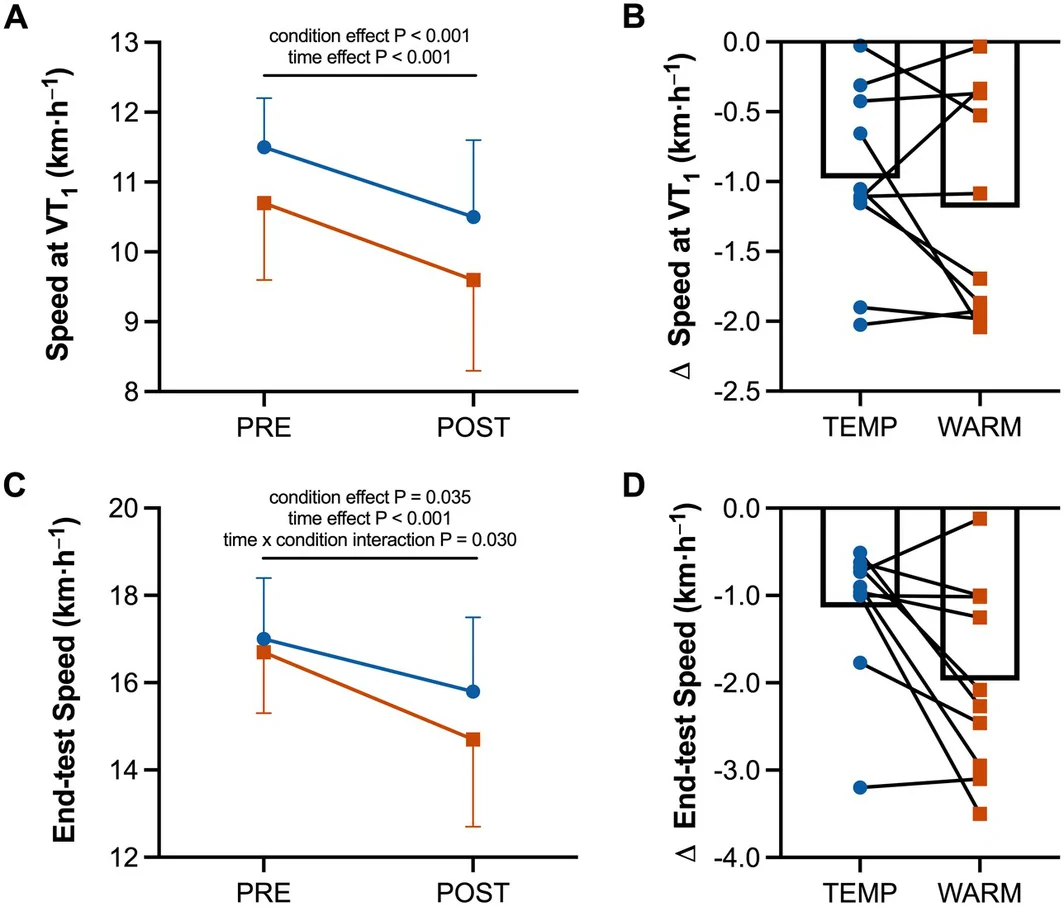
(A) Speed at the first ventilatory threshold (sVT_1_) in temperate (TEMP; blue circles) and warm (WARM; orange squares) conditions before (PRE) and after (POST) 90 min of running; (B) change in sVT_1_ from PRE to POST; (C) end‐test speed in TEMP and WARM before and after 90 min of running; and (D) change in end‐test speed from PRE to POST. Data are presented as mean ± SD in (A) and (C) and as the mean with individual participant data in (B) and (D). *n* = 10.

**TABLE 2:** Physiological variables at V̇O_2_peak and VT_1_ measured before and after prolonged running in temperate (TEMP) and warm (WARM) environmental conditions.

	TEMP			WARM			ANOVA results		
	PRE	POST	Delta (POST − PRE)	PRE	POST	Delta (POST − PRE)	Condition	Time	Time × condition
V̇O_2_peak									
V̇O_2_ (mL·kg^−1^·min^−1^)	56.0 ± 5.7	53.2 ± 6.2	−2.80 ± 4.67	56.8 ± 6.6	54.4 ± 6.4	−2.36 ± 3.11	*F*_(1,9)_ = 1.45 *p* = 0.260 $\eta_{p}^{2}$ = 0.14	***F***_**(1,9)**_ **= 5.89** ***p* = 0.038** $\eta_{p}^{2}$ **= 0.40**	*F*_(1,9)_ = 0.11 *p* = 0.750 $\eta_{p}^{2}$ = 0.01
V̇O_2_ (L·min^−1^)	3.81 ± 0.82	3.44 ± 0.49	−0.37 ± 0.40	3.85 ± 0.82	3.55 ± 0.65	−0.31 ± 0.26	*F*_(1,9)_ = 1.83 *p* = 0.209 $\eta_{p}^{2}$ = 0.17	***F***_**(1,9)**_ **= 11.73** ***p* = 0.008** $\eta_{p}^{2}$ **= 0.57**	*F*_(1,9)_ = 0.48 *p* = 0.505 $\eta_{p}^{2}$ = 0.05
Speed (km·h^−1^)	17.0 ± 1.4^a^	15.8 ± 1.7^a^	−1.14 ± 0.80	16.7 ± 1.4^b^	14.7 ± 2.0^b^	−1.97 ± 1.09	***F***_**(1,9)**_ **= 6.17** ***p* = 0.035** $\eta_{p}^{2}$ **= 0.41**	***F***_**(1,9)**_ **= 37.10** ***p* < 0.001** $\eta_{p}^{2}$ **= 0.80**	***F***_**(1,9)**_ **= 6.59** ***p* = 0.030** $\eta_{p}^{2}$ **= 0.42**
HR (b·min^−1^)	180 ± 16	175 ± 14	−4.80 ± 5.57	183 ± 16	180 ± 13	−3.90 ± 5.43	***F***_**(1,9)**_ **= 7.06** ***p* = 0.026** $\eta_{p}^{2}$ **= 0.44**	***F***_**(1,9)**_ **= 10.81** ***p* = 0.009** $\eta_{p}^{2}$ **= 0.55**	*F*_(1,9)_ = 0.16 *p* = 0.700 $\eta_{p}^{2}$ = 0.02
RER	1.06 ± 0.05	0.96 ± 0.05	−0.09 ± 0.05	1.05 ± 0.05	0.94 ± 0.05	−0.11 ± 0.06	*F*_(1,9)_ = 1.32 *p* = 0.280 $\eta_{p}^{2}$ = 0.13	***F***_**(1,9)**_ **= 39.02** ***p* < 0.001** $\eta_{p}^{2}$ **= 0.81**	*F*_(1,9)_ = 2.65 *p* = 0.138 $\eta_{p}^{2}$ = 0.23
V̇_E_ (L·min^−1^)	140.8 ± 31.0	130.1 ± 31.3	−10.72 ± 13.57	143.3 ± 31.3	125.4 ± 33.7	−17.90 ± 12.72	*F*_(1,9)_ = 0.33 *p* = 0.587 $\eta_{p}^{2}$ = 0.04	***F***_**(1,9)**_ **= 14.95** ***p* = 0.004** $\eta_{p}^{2}$ **= 0.62**	*F*_(1,9)_ = 3.59 *p* = 0.091 $\eta_{p}^{2}$ = 0.29
VT_1_									
V̇O_2_ (mL·kg^−1^·min^−1^)	41.9 ± 3.5	40.3 ± 4.0	−1.66 ± 2.43	39.8 ± 4.1	38.5 ± 4.7	−1.28 ± 2.85	***F***_**(1,9)**_ **= 17.82** ***p* = 0.002** $\eta_{p}^{2}$ **= 0.66**	*F*_(1,9)_ = 4.73 *p* = 0.058 $\eta_{p}^{2}$ = 0.34	*F*_(1,9)_ = 0.15 *p* = 0.706 $\eta_{p}^{2}$ = 0.02
V̇O_2_ (L·min^−1^)	2.84 ± 0.53	2.62 ± 0.46	−0.22 ± 0.17	2.68 ± 0.41	2.51 ± 0.51	−0.16 ± 0.17	***F***_**(1,9)**_ **= 20.01** ***p* = 0.002** $\eta_{p}^{2}$ **= 0.69**	***F***_**(1,9)**_ **= 23.71** ***p* < 0.001** $\eta_{p}^{2}$ **= 0.72**	*F*_(1,9)_ = 0.58 *p* = 0.467 $\eta_{p}^{2}$ = 0.06
Speed (km·h^−1^)	11.5 ± 0.7	10.5 ± 1.1	−0.98 ± 0.65	10.7 ± 1.1	9.6 ± 1.3	−1.19 ± 0.80	***F***_**(1,9)**_ **= 43.80** ***p* < 0.001** $\eta_{p}^{2}$ **= 0.83**	***F***_**(1,9)**_ **= 26.68** ***p*** **< 0.001** $\eta_{p}^{2}$ **= 0.75**	*F*_(1,9)_ = 1.15 *p* = 0.312 $\eta_{p}^{2}$ = 0.11
HR (b·min^−1^)	148 ± 16	145 ± 15	−2.82 ± 6.65	151 ± 17	149 ± 18	−1.39 ± 7.44	*F*_(1,9)_ = 2.94 *p* = 0.121 $\eta_{p}^{2}$ = 0.25	*F*_(1,9)_ = 1.26 *p* = 0.291 $\eta_{p}^{2}$ = 0.12	*F*_(1,9)_ = 0.35 *p* = 0.567 $\eta_{p}^{2}$ = 0.04
EE (kcal·min^−1^)	14.1 ± 2.6	12.7 ± 2.4	−1.42 ± 1.05	13.1 ± 2.1	11.8 ± 2.1	−1.33 ± 0.99	***F***_**(1,9)**_ **= 12.16** ***p* = 0.007** $\eta_{p}^{2}$ **= 0.57**	***F***_**(1,9)**_ **= 24.48** ***p* < 0.001** $\eta_{p}^{2}$ **= 0.73**	*F*_(1,9)_ = 0.08 *p* = 0.789 $\eta_{p}^{2}$ = 0.01
RER	0.87 ± 0.05	0.78 ± 0.03	−0.09 ± 0.06	0.85 ± 0.03	0.76 ± 0.04	−0.09 ± 0.05	***F***_**(1,9)**_ **= 5.88** ***p* = 0.038** $\eta_{p}^{2}$ **= 0.39**	***F***_**(1,9)**_ **= 31.20** ***p* < 0.001** $\eta_{p}^{2}$ **= 0.78**	*F*_(1,9)_ = 0.02 *p* = 0.898 $\eta_{p}^{2}$ = 0.00
V̇_E_ (L·min^−1^)	81.2 ± 18.5	75.0 ± 19.2	−6.23 ± 6.55	77.6 ± 19.9	68.8 ± 17.9	−8.82 ± 8.06	***F***_**(1,9)**_ **= 15.78** ***p* = 0.003** $\eta_{p}^{2}$ **= 0.64**	***F***_**(1,9)**_ **= 16.61** ***p* = 0.003** $\eta_{p}^{2}$ **= 0.65**	*F*_(1,9)_ = 0.85 *p* = 0.381 $\eta_{p}^{2}$ = 0.09

*Note:* Data are presented as mean ± SD. Delta values represent the mean of individual POST − PRE differences. Bold type denotes a significant main effect or interaction (*p* < 0.05). Mass‐specific V̇O_2_ was calculated using body mass measured before prolonged running for PRE values and body mass measured after prolonged running for POST values. Matching superscript letters denote significant difference between PRE and POST within the same condition (*p* < 0.05). *n* = 10.

Abbreviations: EE, energy expenditure; HR, heart rate; RER, respiratory exchange ratio; V̇_E_, minute ventilation.

**FIGURE 5:**
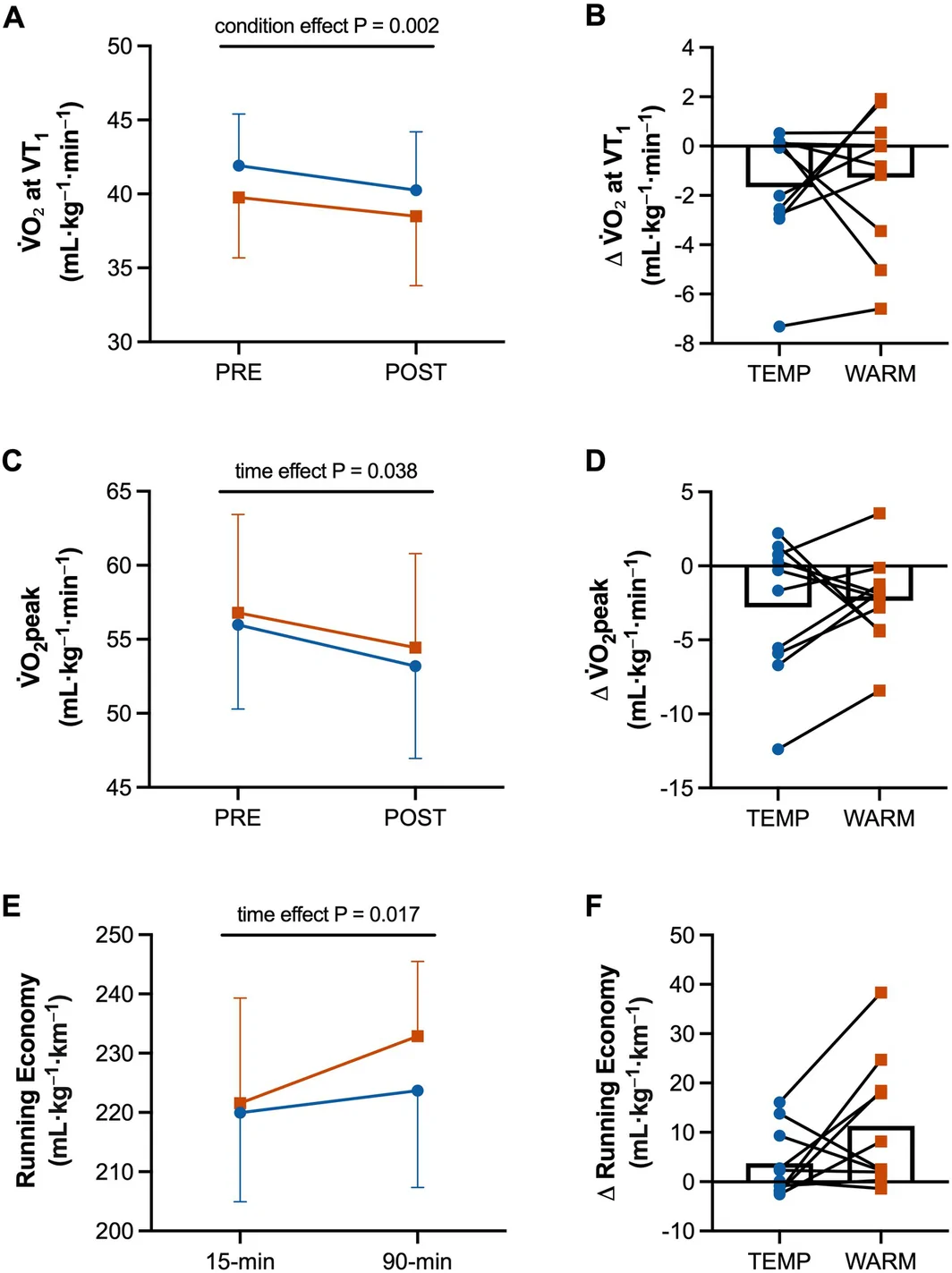
The key determinants of endurance‐running performance in temperate (TEMP; blue circles) and warm (WARM; orange squares) conditions prior to (PRE) and after (POST) 90 min of running: (A) relative oxygen uptake (V̇O_2_) at the first ventilatory threshold (VT_1_), (B) the change in the V̇O_2_ at VT_1_, (C) peak relative oxygen uptake (V̇O_2_peak), (D) the change in V̇O_2_peak, (E) running economy at 15 and 90 min of the prolonged run, and (F) the change in running economy from 15 to 90 min. Data are presented as mean ± SD (A, C, and E) and mean with individual participant data (B, D, and F). *n* = 10.

### 3.3 Maximal Measures

End‐test speed showed a significant effect of time (*F*_1,9_ = 37.10, *p* < 0.001, $\eta_{p}^{2}$ = 0.80), condition (*F*_1,9_ = 6.17, *p* = 0.035, $\eta_{p}^{2}$ = 0.41), as well as significant interaction effects (*F*_1,9_ = 6.59, *p* = 0.030, $\eta_{p}^{2}$ = 0.42). End‐test speed was lower in post vs. pre, and in WARM vs. TEMP, with greater deteriorations evident across time points in WARM (−12.1% ± 6.9%) compared to TEMP (−6.8% ± 5.0%; Figure 4; Table 2). There were significant effects of time for absolute V̇O_2_peak (*F*_1,9_ = 11.73, *p* = 0.008, $\eta_{p}^{2}$ = 0.57) and relative V̇O_2_peak (*F*_1,9_ = 5.89, *p* = 0.038, $\eta_{p}^{2}$ = 0.40), with lower V̇O_2_peak values observed in POST vs. PRE (Figure 5; Table 2). No significant interaction effects or effects of condition were observed for either absolute or relative V̇O_2_peak (all *p* ≥ 0.209). Maximum heart rate was lower in the TEMP vs. WARM (*F*_1,9_ = 7.06, *p* = 0.026, $\eta_{p}^{2}$ = 0.44) and depressed in POST vs. PRE (*F*_1,9_ = 10.81, *p* = 0.009, $\eta_{p}^{2}$ = 0.55), but no interaction effects were evident (Table 2). Both maximal RER (*F*_1,9_ = 39.02, *p* < 0.001, $\eta_{p}^{2}$ = 0.81) and maximal minute ventilation (*F*_1,9_ = 14.95, *p* = 0.004, $\eta_{p}^{2}$ = 0.62) decreased from pre to post, with no differences evident between conditions and no significant interaction effects (Table 2).

## 4 Discussion

This is the first study to determine whether environmental heat stress compromises durability of the primary physiological determinants of long‐distance running performance. The key findings from the study are that: (i) in the rested state, warm conditions of 30°C and 40% relative humidity lowered sVT_1_ by ~7%, and lowered relative V̇O_2_ (~5%) and metabolic energy expenditure (~7%) at VT_1_, compared to temperate conditions; (ii) 90 min of running reduced sVT_1_, absolute V̇O_2_ at VT_1_, V̇O_2_peak, and end‐test speed in both conditions; and (iii) there was no statistical evidence that WARM altered the decline in sVT_1_ or V̇O_2_peak, although RE and end‐test speed deteriorated to a greater extent in WARM.

While there was no statistical evidence that WARM altered the magnitude of decline after 90 min of running in TEMP and WARM conditions, key distance‐running markers in WARM were already compromised before the prolonged run. Therefore, the observed percentage declines (sVT_1_: −8.6% in TEMP, −11.1% in WARM) were superimposed on a lower baseline performance, leading to a greater absolute reduction in performance compared with the optimal conditions observed before prolonged running in TEMP, despite participants running at a slower speed in WARM. The sVT_1_ measured in a fatigued state in WARM was, on average, 17.1% below the value obtained rested in TEMP (9.6 vs. 11.5 km·h^−1^). Therefore, warm conditions and prior exercise combined to place runners at a substantially lower absolute physiological capacity, even when the prolonged run was prescribed relative to condition‐specific capacity. Importantly, we compared durability responses to exercise between environments at matched relative intensities, and therefore lower absolute speeds under heat stress. At 15 min, participants were running at a similar percentage of V̇O_2_ at VT_1_ in TEMP (103.4 ± 4.1%) and WARM (102.1% ± 3.0%), confirming that relative intensity was matched relative environments. This approach reflects how many runners respond in competition, as marathon performance slows as environmental temperature rises [39]. However, some runners may initially maintain target paces established under cooler training conditions rather than adjusting sufficiently for the heat [40], while pacing in championship races is often shaped by tactical behavior rather than physiological capacity alone [41]. The conditions were moderately warm rather than hot, similar to warm spring or autumn racing in a temperate climate, and these findings may not extend to more severe heat or humidity.

### 4.1 Moderate‐To‐Heavy Transition

Warm conditions lowered the moderate‐to‐heavy transition before prolonged running. In the rested state, the speed at VT_1_ was ~0.8 km·h^−1^ (~7%) lower in WARM, accompanied by lower V̇O_2_ and metabolic energy expenditure at VT_1_. This aligns with prior running [1] and cycling [10, 11] studies. In keeping with previous findings [20, 21, 22, 23], the speed and V̇O_2_ at VT_1_ declined during prolonged running. However, the condition × time interaction for sVT_1_ was not statistically significant. On average, sVT_1_ fell by 0.21 km·h^−1^ more in WARM than TEMP, but with wide 95% CI spanning −0.65 to +0.23 km·h^−1^. These data therefore neither show additional deterioration in WARM nor establish equivalent durability between environments.

The absence of a statistically significant interaction for sVT_1_ may be partly explained by reductions in metabolic energy expenditure at VT_1_ in both conditions. Barrett and Maunder [20] reported that the reduction in speed at the moderate‐to‐heavy transition following prolonged running was attributable to a fall in metabolic power rather than deterioration in RE. In the current study, the rate of metabolic energy expenditure at VT_1_ fell by ~10% in TEMP and WARM, suggesting an inability to sustain the same metabolic rate at the moderate‐to‐heavy boundary. Although RE during the prolonged run deteriorated more from 15 to 90 min in WARM (5.4% ± 6.4%) than TEMP (1.7% ± 3.1%), this difference may not have been large enough to produce a statistically greater reduction in sVT_1_. Therefore, the decline in metabolic power appears to have been the dominant contributor to the fall in sVT_1_ across conditions.

Another potential contributing factor to the lack of interaction effects could be the experimental design. The prolonged run was prescribed relative to condition‐specific PRE measures, at the same relative intensity, so participants ran more slowly (11.0 ± 1.1 vs. 11.8 ± 0.7 km·h^−1^), covering less distance (16.5 ± 1.6 vs. 17.7 ± 1.1 km), and expending less energy (1228 ± 196 vs. 1300 ± 223 kcal) in WARM compared to TEMP. This design choice reflects the practical situation in which runners typically reduce pace in warm environments, but it also means that the external workload was lower in WARM. Since exercise intensity is an important determinant of durability [42], the lower absolute workload in WARM may have reduced the potential for additional deterioration.

### 4.2 Running Economy

RE deteriorated during prolonged running, and the pattern differed between conditions. Although post hoc comparisons did not identify individual time points that differed from the 15‐min baseline, the significant condition × time interactions for RE and EC indicate that warm conditions altered the temporal response of RE. Previously, mixed findings have been shown for RE during prolonged running, with deterioration evident [23], or not apparent over time [20, 21, 22]. Evidence in warm or hot running conditions remains limited, although cycling data suggest that oxygen cost can increase more over time in the heat than in cooler conditions [14].

Changes to whole‐body substrate use during heat stress did not appear to explain the worsening of RE. Despite running at significantly different speeds, both conditions exhibited similar carbohydrate and fat oxidation rates (Figure 3C,D). Absolute energy expenditure was, however, lower in WARM, and carbohydrate accounted for a similar share of total energy use in each condition (~46% in TEMP and ~44% in WARM). This contrasts with the findings of a systematic review and meta‐analysis by Mougin et al. [43], which showed that carbohydrate use was increased during prolonged exercise in hot conditions. The discrepancy may partly reflect differences in exercise prescription because many of the included studies matched absolute workloads across conditions, whereas the present study matched workload relative to condition‐specific capacity. In any case, a greater reliance on carbohydrate would be expected to improve, rather than worsen, RE, and so seems an unlikely candidate for the worsened RE in WARM. Blood lactate was not measured, however, so we cannot partition the relative contributions of oxidative and non‐oxidative pathways, and the oxygen cost of running captures only the oxidative component. Irrespective of mechanism, the greater deterioration in RE in WARM means that a given speed would require a greater fraction of V̇O_2_peak late in a race in the heat. This may partly explain why end‐test speed fell more in WARM despite a similar decline in V̇O_2_peak and suggests that RE measured in a fresh state may underestimate the oxygen cost of running late in warm‐weather races.

Several mechanisms may have contributed to the altered RE response. Ventilatory demand is one candidate, as hyperthermia increases ventilation during prolonged exercise [17], and minute ventilation rose to a greater relative extent in WARM than TEMP. However, the oxygen cost of ventilation is modest at submaximal intensities [44], so it is unlikely to account for most of the deterioration in RE. Indeed, when modeling the relationship between ΔV̇O_2_/ΔV̇_E_ using the slopes reported by Vella et al. [44], the extra V̇_E_ between 15 and 90 min accounted for an additional 10.5 ± 7.0 mL·min^−1^ V̇O_2_ in TEMP and 13.9 ± 10.0 mL·min^−1^ V̇O_2_ in WARM. Therefore, other factors, including muscle temperature, cardiovascular drift, neuromuscular recruitment, glycogen availability, metabolite accumulation, and excitation‐contraction coupling, are likely to have contributed to a greater extent, but these remain speculative and require targeted investigation.

### 4.3 Peak Oxygen Uptake and End‐Test Speed

V̇O_2_peak declined from PRE to POST in both conditions. Absolute V̇O_2_peak declined from 3.81 ± 0.82 to 3.44 ± 0.49 L·min^−1^ (−8.3%) in TEMP and from 3.85 ± 0.82 to 3.55 ± 0.65 L·min^−1^ (−7.4%) in WARM. Relative to body mass, V̇O_2_peak declined from 56.0 ± 5.7 to 53.2 ± 6.2 mL·kg^−1^·min^−1^ (−4.8%) in TEMP and from 56.8 ± 6.6 to 54.4 ± 6.4 mL·kg^−1^·min^−1^ (−4.0%) in WARM. However, there was no statistical evidence that WARM altered the magnitude of this decline. This is noteworthy given the previous association between cardiovascular drift and reductions in V̇O_2_max [3, 5, 45]. However, in the present study, the heat stimulus was moderate, participants began the incremental tests shortly after entering the chamber, and V̇O_2_peak did not differ between TEMP and WARM in the rested state. The absence of a condition effect on PRE V̇O_2_peak may therefore reflect the limited initial thermal strain and the moderate environmental conditions used here, rather than evidence that heat stress has no effect on maximal aerobic capacity. This is consistent with findings showing larger heat‐related reductions in V̇O_2_max when exercise is performed in more severe conditions [45, 46] or after greater pre‐test thermal strain [4, 47] when compared to the current study.

Like the moderate‐to‐heavy transition, a possible explanation for the lack of significant interaction effect is likely the intensity‐matched design. Previously, in cycling, the durability of V̇O_2_peak has been shown to be dependent on the intensity domain in which the prolonged bout of exercise is performed [48]. Further to this, V̇O_2_peak also did not differ between conditions in PRE, so the absence of an interaction may reflect the limited severity of heat stress rather than durability itself. Indeed, several previous studies that have observed a decrease in V̇O_2_max in hot conditions have used more challenging environmental conditions (e.g., 35°C, 40°C, and 45°C, 50% RH [4]; 32°C, 60% RH [1]; 35°C, 40% RH [45]). The percentage change in V̇O_2_max relative to control conditions appears to act in a dose‐dependent manner, in which greater deterioration is observed at higher temperatures [4]. It could be posited that the environmental stimulus used in the current study was not sufficient to elicit a significant response in V̇O_2_peak. Nonetheless, end‐test speed decreased following prolonged running to a greater extent in WARM compared to TEMP (~12.1% vs. ~6.8%, respectively; Table 2), indicating peak running speed may deteriorate further in warm conditions. This may reflect the greater deterioration of RE observed in the WARM condition, whereby the body's ability to convert a similar V̇O_2_peak into locomotion was shown to be impaired. This is consistent with previous research which demonstrates a disproportionately larger decrease in the end‐test speed when compared to changes in V̇O_2_max [4].

### 4.4 Limitations

Several methodological factors should be considered when interpreting these findings. First, the prolonged running bout was prescribed relative to condition‐specific physiological capacities. As a result, participants ran at a lower speed, covered less distance, and expended less energy in WARM compared to TEMP. Future studies should compare both relative and absolute workload‐matched designs to examine the effects of heat stress when the external demands are the same. Second, the environmental stimulus represented a moderate heat stress, and the present findings may not generalize to hotter or more humid environments. Third, although several physiological and perceptual responses in both conditions were characterized, direct measures of cardiovascular function, neuromuscular fatigue, muscle temperature, glycogen availability, and muscle metabolism were not obtained. Similarly, we did not log inspired oxygen fraction continuously, so we cannot rule out a progressive fall in chamber oxygen during the prolonged trials. The chamber was ventilated throughout testing, so any depletion was likely offset and seems an unlikely source of meaningful error. Any underestimation of V̇O_2_ would apply equally in WARM and TEMP, so the within‐participant durability comparisons are unlikely to have been distorted. Hydration status was not formally quantified either before or after the prolonged trials. Participants were asked to arrive having followed standardized food and fluid intake, and urine osmolality was screened on arrival, but neither a baseline euhydrated body mass nor 24‐h urine collection was obtained. We therefore cannot confirm that participants began each trial euhydrated, and any small differences in pre‐trial hydration between visits cannot be excluded. As hydration status was not formally quantified or controlled, we cannot fully separate the effects of heat from those of hypohydration. Body mass losses were greater in WARM (−3.5% ± 0.9%) than TEMP (−2.7% ± 0.8%), and hypohydration adds to cardiovascular and thermal strain [49]. Some of the greater deterioration in RE and end‐test speed in WARM may therefore reflect the combined effects of heat and fluid loss, rather than heat alone. Fourth, water was provided at ~37°C to minimize artifacts in gastrointestinal temperature measurement following fluid ingestion. However, cooler fluid is more palatable during exercise, and the use of warmed water may have suppressed ad libitum fluid intake. This may have contributed to the body mass losses observed during the prolonged trials, particularly in WARM, and should be considered when interpreting the magnitude of sweat‐related body mass change. Further work combining durability assessments and these measures would provide greater mechanistic insight. Fifth, durability was quantified as the PRE‐to‐POST change in each determinant, and the reliability of such changes may be considerably lower than that of the absolute measurements. Absolute sVT_1_ has shown good‐to‐excellent reliability both before and after prolonged running (ICC ~0.92–0.95), whereas its percentage deterioration has shown poor reliability (ICC ~0.07–0.25), with a typical error of ~3.6–3.9 percentage points [32]. These estimates were obtained using a different exercise protocol and cannot be transferred directly to the present study. Nonetheless, they suggest that measurement error in the change scores may have limited our ability to detect modest between‐condition differences in durability. Finally, only 10 participants out of the 15 initially recruited completed the study. Of these, two participants were unable to complete the prolonged trial in WARM, despite completing this in TEMP. Therefore, the results presented may not represent the full effects of warm conditions in runners with limited heat tolerance.

## 5 Conclusion

Prolonged running reduced several determinants of performance, while warm conditions lowered baseline threshold‐related and performance‐related measures. Specifically, sVT_1_ was lower in WARM than TEMP and declined following 90 min of running in both conditions. Similarly, V̇O_2_peak was reduced following prolonged exercise, irrespective of environmental condition. In contrast, RE exhibited a different response between conditions over time, deteriorating to a greater extent during prolonged running in WARM than TEMP. Despite this, we found no statistical evidence that heat altered the magnitude of the decline in the moderate‐to‐heavy transition or V̇O_2_peak following prolonged exercise when exercise intensity was prescribed relative to condition‐specific physiological capacity. Nevertheless, the combined effects of warm conditions and prior exercise resulted in substantially lower absolute physiological capacity, with sVT_1_ being ~17.1% lower following prolonged running in WARM compared with a rested state in TEMP. Furthermore, end‐test speed deteriorated to a greater extent in WARM, suggesting that maximal running performance may be more sensitive to the combined effects of thermal strain and prior exercise than V̇O_2_peak alone. Collectively, these findings indicate that runners should adjust pace according to environment‐specific physiological capacity. Future investigations are warranted to understand whether interventions (e.g., heat acclimation, precooling, nutritional supplementation) can mitigate against the negative outcomes observed in the current study.

## 6 Perspective

We demonstrate that warm conditions reduce the absolute physiological capacity available to runners and can exacerbate the deterioration in RE during prolonged exercise. However, when exercise intensity was prescribed relative to condition‐specific physiological capacity, we found no statistical evidence that warm conditions altered the magnitude of decline in threshold‐related or maximal aerobic measures. These findings suggest that the issue facing runners in warm environments may be less about accelerated deterioration of physiological parameters and more about operating from a lower physiological baseline while experiencing a greater deterioration in RE over time.

## Funding

Ben Hunter received funding from The Great Britain Sasakawa Foundation to support a research visit to the University of Tsukuba with Naoto Fujii, which facilitated collaboration related to this study.

## Conflicts of Interest

The authors declare no conflicts of interest.

## Data Availability

The data that support the findings of this study are available from the corresponding author upon reasonable request.
